# Are We Truly Winning the War Against Malnutrition? A Review of *Within Our Grasp* by Sharman Apt Russell and Conversations with Terrie Taylor, Natasha Hochberg, and David Hamer

**DOI:** 10.4269/ajtmh.22-0006

**Published:** 2022-03-14

**Authors:** Claire Panosian Dunavan

**Affiliations:** UCLA Division of Infectious Diseases CHS 52-215, 10833 Le Conte Avenue Los Angeles, California E-mail: cpanosian@mednet.ucla.edu

It’s Summer 1972. I’ve just finished college and I’m spending the summer in Haiti. When I finally reach l’Hôpital le Bon Samaritain after an 8-hour journey in a rickety *camion*, I know nothing about malnutrition. One month later, seeing toddlers with oddly red hair, cracked skin, and swollen limbs no longer shocks me. Nor am I shocked that some of them never return home.

Malnutrition, an age-old killer and accomplice in the deaths of impoverished children, is my first introduction to tropical medicine.

Seven years later, I attend the London School of Tropical Medicine and Hygiene and take a deeper dive into malnutrition’s history, physiology, and perilous synergy with infectious diseases. Nonetheless, my fellow students and I are still “looking through a glass darkly.”

Then come science and strategies that fuel hope. When experts in the 1980s and 1990s champion micronutrients plus diverse, balanced diets; WHO’s initial package of Expanded Programme on Immunization vaccines; and oral rehydration for childhood diarrhea, child survival rises. Over time, re-feeding regimens meant to rescue children from death are replaced by ready-to-use therapeutic foods, which often forestall it.

Today, global experts also parse proximate causes of malnutrition from colonialism to climate to monoculture, as well as lifelong consequences such as stunting.^1^ Plus, they have a better handle on burden. Sadly, in 2020, we backslid. During the first year of the COVID-19 pandemic, the United Nations estimates roughly 150 million children under 5 were stunted, 45 million were wasted,^2^ and more than 800 million people were hungry. A year later, as the WHO Director for Nutrition and Food recently told *The Lancet,* global food insecurity had continued to grow and worsen malnutrition. So is the glass half empty or half full? And how should a public-facing author weigh discouraging facts against the past three decades’ otherwise quiet-but-steady progress?

In *Within Our Grasp—Childhood Malnutrition Worldwide and the Revolution to End It,* Sharman Apt Russell weaves modern nutritional science with interviews and blueprints for hope. Looking back, what especially inspires hope are stories of people determined to fight malnutrition, like those who developed the nutrient-rich paste Plumpy’Nut (in Malawi, it is called Chiponde). Does Russell’s book also carry a whiff of cultural tourism? Yes. But, to me, this was far outweighed by the book’s sincere and illuminating conversations with newly empowered farmers, organizers, and teachers (almost all of them women) during the author’s 2016 field trip to Malawi.

“Russell’s passion for citizen science and her jargon-free presentation of information relating to malnutrition will open worlds for most readers, from high school students to sociologists” is how *Library Journal* describes *Within Our Grasp*, and that’s just one of many glowing blurbs on Amazon. A professor emeritus in humanities at Western New Mexico University and associate faculty at Antioch University, Russell previously authored *Diary of a Citizen Scientist* (winner of the 2016 John Burroughs Medal), *Knocking on Heaven’s Door* (winner of the Arizona/New Mexico Book Award), *Teresa of the New World* (winner of the Arizona Authors Association Award), and (with funding from the Rockefeller Foundation) *Hunger: An Unnatural History.*

In short, *Within Our Grasp* is an excellent book to recommend to undergrads majoring in global development, students of medicine and public health, and post-graduate trainees embarking on overseas rotations in low- and middle-income countries. Or anyone else, for that matter, who wants to explore modern solutions to the age-old problems of hunger and survival of the weakest.

After finishing *Within Our Grasp,* I turned to three American Society of Tropical Medicine and Hygiene colleagues for further perspective. For 35 years, Dr. Terrie Taylor has done malaria research in Malawi, where Russell did her grassroots reporting. Dr. Natasha Hochberg currently studies the nexus of undernutrition and tuberculosis in India. Dr. David Hamer’s work is largely focused on implementation research designed to advance maternal, newborn, and child health in real-life settings in low- and middle-income countries.

## INTERVIEW WITH DR. TERRIE TAYLOR^3^

### Looking back on your decades in Africa, please describe your earliest experiences of malnutrition.

My earliest research project in Africa involved onchocerciasis in Sudan. I was mainly based in Khartoum, but the field sites were up north in a place called Abu Hamad and in southern Sudan (not yet a country) in a town called Wau. As far as I could tell, those folks were not suffering from malnutrition. I wouldn’t say it was a time of plenty—they had fairly monotonous diets—but there was plenty of food.

Then I went to Malawi in July 1986, and it was a much more appealing place to work. There were roads! There was electricity! You could imagine functioning there. Of course, it was also the best time of year to be there. The harvest had just finished, so everybody had food. I was still hoping to study onchocerciasis, but when I met the only pediatrician at Queen’s Hospital in Blantyre, she thought I should I focus on malaria, which was the single highest cause of admissions to the hospital for kids and the highest cause of death. So, I began my work on malaria.

But once the medical school opened in 1991, there was “handover,” or morning report. So I would go to the morning report and listen to this litany of how many kids were admitted yesterday, which wards they went to, and how many died.

Malnutrition had, far and away, the highest mortality rate: 30%, 40%, compared with 10% in other sections of Pediatrics. The malnutrition wards were overflowing with marasmic and kwash kids. Overflowing.

### And this was just the tip of the spear, right?

Yes, it was the early nineties, and it was grim. As Sharman Apt Russell describes in her book, there were people trying many different approaches to malnutrition without really understanding it. But slowly there were breakthroughs in management.

I remember when Mark Manary came to Malawi as a proponent of Project Peanut Butter. He was a quiet, serious person who did a ton of work and built this factory, but you had to pry the story out of him. He was never showy; it was never about him.

He had just started to encourage community-based management of malnutrition as opposed to hospital-based care. It was much more sustainable, much more cost-effective.

### What about the situation today?

I double-checked with the head of Pediatrics. Now, the kids who come into the hospital for malnutrition are those with co-morbidities, like cerebral palsy, or very bad birth injuries. The hospital doesn’t see “regular” malnutrition any more.

I also asked about possible bias because I have always worked in the central hospital in a big city as opposed to a rural setting, but he said the improvement in nutritional status was evident at all levels of the health-care system. Then I discovered a very nice survey that covered 10,000 kids in 10 districts—rural and urban. Rates of child malnutrition are now at an all-time low of 5%. There was a little blip with COVID and the food insecurity associated with it, but the overall situation is so much better. The messages about balanced diets are getting through, and Chiponde is available for free through the Ministry of Health. That’s something else I wanted to emphasize. Malawi has been unusual on the continent in hewing to free health for all.

It’s true there are two parallel health systems—private and public—but the public system works. The Chiponde gets out to the health centers, and kids who need it come and pick it up on a regular basis and take it home. It’s also logged in. People keep track of it.

### Do you recall Malawi’s food crisis of 2002?

Although it had been building since the bad rains of the previous year, it hit very quickly. The cassava failed, and then the maize stocks were sold from Malawi. So it was a juxtaposition of adverse weather and bad political decisions followed by no communication. Then, all of a sudden, we realized, “Oh my God. There’s no food.” Malawi had already fallen from grace with the Danes and the Brits, so it wasn’t easy to generate external support.

I was there just six months, and the malaria season was rough, but the famine lasted until the crops came in. At that point, things began to improve, and there was also a change in government. Against international advice, the next president invested in fertilizers. Within two or three years, maybe by 2005 or 2006, Malawi was exporting maize.

### What can you share about climate and agricultural development in Malawi?

With respect to climate, people are starting to notice that everything is exaggerated. The cold season is colder. The rainy season is wetter. But beyond that . . . it’s kind of like what’s been happening in the U.S. “Oh, this is the 10th hundred-year storm.” So awareness of climate change is just beginning to dawn.

In terms of agriculture and technology, Africa is definitely embracing more technology that enhances productivity . . . in fact, the benefits of that technology probably outweigh the negative impacts of weather. Right now, for example, there’s not a lot of pesticide use, but the land is being flogged with fertilizer.

### Let’s finish with Russell’s book. What about it impressed you most?

What impressed me most were the stories of the community-based workers who hung in there through thick and thin. And the well-observed details. It also made me go back in time and think. I’ve seen three and a half decades of attempts to use a variety of community-based approaches in Malawi. People just kept at it. This is how long it takes to see results. We’ve seen it with malaria, we’ve seen it with maternal mortality rates, and we’ve seen it with malnutrition.

## INTERVIEW WITH DR. NATASHA HOCHBERG^4^

### I realize you often witnessed the intersection of poverty and infectious diseases before launching your current line of research. What was the moment of epiphany that led you to focus on malnutrition and tuberculosis?

Yes, while working internationally in places like Peru, Honduras, Bangladesh, Niger, and Togo, the intersection of poverty and infectious diseases was always apparent to me. But my research focusing on undernutrition and TB [tuberculosis] began roughly 10 years ago at a meeting in India where TB researchers were talking about designing novel diagnostic tests and improving adherence to treatment, and a leader in the field made a powerful statement. In effect, Anurag Bhargava said, “You’re missing the picture. Malnutrition is really the leading driver of TB worldwide.” His talk had a profound effect on me as I thought, *Why put so much emphasis on understanding the intersection of HIV and TB, or diabetes and TB, or evaluating new diagnostics, while paying so little attention to the role of undernutrition?* From a research perspective, there are so many questions to answer. And from a public health perspective, there’s so much we need to do.

### Please continue with further background on TB and undernutrition.

First of all, with 10 million cases and 1.5 million deaths in 2020, TB remains the leading infectious killer worldwide after COVID-19. But what we now know is that a quarter of TB cases worldwide, and, in some parts of the world, more than 50% of cases are attributable to malnutrition. That in itself is striking, but there are actually many ways in which malnutrition and TB intersect. Malnutrition not only increases the risk of developing active TB disease, but also drives poor outcomes. Malnourished people more often fail therapy, which increases onward transmission and places them at a heightened risk for drug-resistant infections. Finally, they more often develop severe disease, and they’re more likely to die of TB.

We now have data from our Indian cohorts that correlate malnutrition and mycobacterial burden and patients’ radiographic extent of disease. In one of our studies, TB patients with severe malnutrition had, on average, 11% more lung affected compared with those with a normal body mass index, and they also had an almost 5-fold increased odds of pulmonary cavitation. This reinforces the idea that malnutrition affects the pathogenesis of disease. Malnutrition also affects the pharmacokinetics and absorption of TB drugs. This has been demonstrated by data from our colleagues showing low levels of rifampicin and isoniazid in malnourished children. So now we’re starting to move to population-level work. What can be done about this? Our research group recently completed modeling work which suggests that nutritional interventions could be a cost-effective way to prevent TB disease in India; this paper has now been published in *Clinical Infectious Diseases*.

### Can you review some historical data that buttress the case for decreasing TB disease by augmenting nutrition?

One example is the Papworth Village Settlement in England in the pre-antibiotic era of the early 1900s, where TB patients received food, housing, employment, and medical supervision. Anurag Bhargava and his team went back and analyzed its data to compare the rates of disease among family members of Papworth residents with rates among contacts of TB patients in surrounding areas. Although the Papworth interventions did not reduce TB transmission (children still became infected), they markedly reduced progression to TB disease. Other data come from a World War II prisoners of war camp in Germany, where they looked at rates of TB disease among different groups of soldiers, some of whom received a 1,000 calorie-per-day Red Cross supplement in addition to the standard camp diet. Those who did not receive the additional protein and calories had much higher rates of TB disease.

### Do your current studies in India include children?

Unfortunately not yet, partly because diagnosing TB in children is tricky. It’s not impossible, but it requires a whole different algorithm. That’s why our current work is primarily focused on adults. At the same time, my hope is eventually to move into the world of kids, not only because malnutrition increases their risk for many respiratory infections, but because they’re also at higher risk than adults for progressing to active TB following exposure. My ultimate goal is to encourage public health programs to build inter-sectoral collaborations and integrate nutrition in the fight against TB and other infectious diseases.

## INTERVIEW WITH DR. DAVID HAMER^5^

### David, what initially inspired your interest in child malnutrition?

On my first day as an undergraduate at Amherst, I met a friend who later repeatedly said, “You need to go to Bangladesh. My country needs people to work in it.” And so I eventually went to Dhaka during medical school and did research at the Institute for Food Science and Nutrition. I also spent time at the International Centre for Diarrhoeal Disease Research (icddr,b) and rotated through a clinical unit where I was floored to see so many children with marasmus and kwashiorkor, and eyes completely destroyed by a lack of vitamin A. I also saw a variety of infections superimposed on wasting and stunting. That’s what triggered my interest.

### Fast-forward to Zambia.

In 2011, I went to Zambia because I had just received a ten-million-dollar grant from the Gates Foundation for a community-based intervention using chlorhexidine for newborn cord care. But being in Zambia was like being in a public health grocery store. There were just so many things to pull off the shelf and work on. Among other things, I had the opportunity to evaluate the impact of a nationwide program of rural sanitation and hygiene on wasting and stunting.

### Which brings us to the contribution of wasting and stunting to childhood disease. Can you summarize some key takeaways from a recent Pneumonia and Wasting scorecard?^6^

Yes. In 2019, wasting, which is the leading risk factor for child pneumonia deaths, contributed to 55% of deaths. In addition, 90% of wasting-related child pneumonia deaths occurred in 40 low- and middle-income countries, the majority in Africa. So even though wasting accounts for a relatively small proportion of undernutrition compared with underweight or stunting, when it’s present, it’s a major risk factor both for acquiring infection and for more severe cases of pneumonia and diarrhea. In malaria, the data are more complicated, but wasting probably increases its severity as well. So we really need to address wasting to reduce both the burden and mortality of infectious diseases in younger children.

Stunting, on the other hand, is a different problem because it’s so widespread and multifactorial. Some of it may start in the antenatal period, or even pre-conception. When that happens, children are small for gestational age at birth. But most stunting results from what happens to a child during their first year or two of life. This includes inadequate nutrition and dietary protein, and probably micronutrients like zinc that are important for growth. In addition, inadequate hygiene, poor sanitation, and contaminated water leading to food- and waterborne infections and diarrhea, all contribute to stunting.

### What are some possible ways to reduce stunting?

For several years now, we’ve been looking at one particular strategy in Zambia. In fact, we just completed our data collection in rural areas of Eastern and Southern Province. Although I sometimes call it a pilot study, we actually started with a small, randomized controlled trial of community-based mothers’ groups (except that they’re now called parenting groups because we’re trying to involve fathers as well) where we enrolled mothers with children who were six months or younger. We picked one mother to be the leader and gave her a curriculum. Every two weeks, the mothers met and received education in a fun, interactive way that included games, challenges, and practical teaching about age-appropriate nutrition. That meant we emphasized breastfeeding during the first six months of life; then safe complementary foods; then dietary diversity using low-cost, locally-available sources of proteins such as ground nuts (peanuts) or, in Zambia, little baby fish called *kapenta*, because protein-calorie malnutrition is a major factor in stunting. We also encouraged adding locally available fruits and vegetables that mothers might not otherwise use.

Our goal over time was to improve nutritional status, growth, and cognitive development. Our 2014–2016 study of 500 children in southern Zambia accomplished all three goals. So, for the last two and a half years, we’ve been doing the same thing on a larger scale with 5,000 mother–baby pairs along with a detailed evaluation of a subset, because we don’t need the full 5,000 for adequate power. It will be interesting to see if our latest data replicate our previous results, because the more recent program was integrated into public health plus community-based organizations.

### That sounds great. Who’s been funding these efforts?

The funding came primarily from USAID Zambia, but also Grand Challenges Canada, which had a program called Saving Brains. That just ended, but USAID subsequently funded an offshoot emphasizing child development. Fortunately, my team includes two people with this expertise. A book of Zambian folk stories has now been translated into multiple Bantu languages to evaluate our program’s impact on older children from some of our households with respect to their ability to read.

### What can you share about the recent impact of COVID-19 on food security and dietary diversity in Africa?

Although I wasn’t directly involved in the study, I served as an associate editor for a collection of papers done by ARISE, a network of collaborators in Ethiopia, Nigeria, and Burkina Faso, and published in 2021 in the *American Journal of Tropical Medicine and Hygiene*. One of the leaders of ARISE is Wafaie Fawzi at the Harvard School of Public Health. During the early part of the pandemic, ARISE conducted a series of rural and urban surveys using cell phones. One high-level takeaway in 1,800 households was COVID-19’s negative effects on the price of food and dietary quality. Dietary diversity and prime diet quality scores also fell. This was partly due to higher prices, but also to decreased access to food.

### Let’s end on the future of community versus hospital-based management of child malnutrition.

For years, a lot of acute malnutrition was managed at the hospital level. But in recent years, there’s been a push to see if some management can be offloaded to the primary health center or the community. For moderate acute malnutrition, this is entirely feasible. For milder cases or variants of severe acute malnutrition, it’s also possible, but first you need screening procedures. Then you need referral systems so children can be adequately assessed to determine whether they can be safely managed at home as opposed to a health facility. You also need people trained to monitor the children and provide appropriate supplements.

Basically, a lot of my work over the last 20 years has involved testing health interventions that are already backed by science. But we still need to figure out how to make them happen in real-world settings. Once that becomes clear, we can develop better guidelines and implementation strategies.
